# Evaluation of Survival Rate and Associated Factors in Patients with Cervical Cancer: A Retrospective Cohort Study

**DOI:** 10.34172/jrhs.2022.87

**Published:** 2022-07-11

**Authors:** Fatemeh-Sadat Tabatabaei, Arefeh Saeedian, Amirali Azimi, Kasra Kolahdouzan, Ebrahim Esmati, Afsaneh Maddah Safaei

**Affiliations:** ^1^Radiation Oncology Research Center, Cancer Research Institute, Tehran University of Medical Sciences, Tehran, Iran; ^2^School of Medicine, Tehran University of Medical Sciences, Tehran, Iran; ^3^Department of Radiation Oncology, Cancer Institute, Imam Khomeini Hospital Complex, Tehran University of Medical Sciences, Tehran, Iran

**Keywords:** Neoplasm Staging, Survival Analysis, Adjuvant Radiotherapy, Uterine Cervical Neoplasm

## Abstract

**Background:** Cervical cancer, the most common gynecological cancer, is a matter of concern, especially in developing countries. The present study investigates survival rates, associated factors, and post-treatment follow-up status in cervical cancer patients.

**Study Design:** A retrospective cohort study.

**Methods:** This study was conducted on 187 patients referred to an academic referral cancer center in Iran from 2014-2020. Overall survival (OS) and event-free survival (EFS) were evaluated using Kaplan Meyer analysis. The event was defined as recurrence, metastasis, or death.

**Results:** The patients came for post-treatment visits for a median of 36 months (interquartile range [IQR]: 18-51). The median OS and EFS were 24 and 18 months, respectively. The 1- and 3- year OS rates were 90% and 72%, respectively. The 1- and 3- year EFS rates were 76% and 61%, respectively. Stage≥III (hazard ratio [HR]: 3.1, 95% confidence interval [CI]: 1.5, 6.5, *P*<0.001) and tumor size>4 cm (HR: 2.5, 95% CI: 1.2, 4.9, *P*=0.006) predicted lower OS. The most common histopathology was squamous cell carcinoma (SCC) (71.1%) with non-significant higher 3- year OS (HR: 0.62, 95% CI: 0.33, 1.16, *P*=0.13). No significant difference in OS was found between adjuvant and definitive radiotherapy in both early and advance-staged patients (Log-rank=0.7 *P*=0.4, log-rank=1.6, *P*=0.2, respectively).

**Conclusion:** As evidenced by the obtained results, the survival of patients was lower compared to that in developed countries. Higher stage and tumor size led to shorter survival. The histopathology and type of treatment in comparable stages did not have any significant impact on survival.

## Background

 Cervical cancer is the fourth most common cancer in women after breast, colorectal, and lung cancers.^1,2^ Based on the most recent worldwide analysis of cervical cancer, in 2020, about 604 000 incident cases and 341 000 attributable deaths were reported, with the highest rates in countries with low socio-demographic index.^3^ Cervical cancer survival rates differ commonly based on the country’s development status. Approximately 84% of all cervical cancer cases and 88% of cervical cancer deaths occur in developing countries.^1^ Among developed countries, the United States and England have a five-year survival rate of more than 65% and 60%, respectively.^4,5^ On the other hand, in developing countries, such as Thailand, a five-year survival rate exceeded 50%.^6^ In Iran, based on International Agency for Research on Cancer (IARC), the age-standardized incidence and mortality rates in 2020 were 7.3 and 1.5 per 100 000, respectively.^3^

 According to the National Comprehensive Cancer Network (NCCN), invasive cancers in the early stages of the disease can be treated by radical hysterectomy or radiotherapy, and locally advanced cancers are treated with definitive radiotherapy.^7-10^ Several studies have been carried out to explore factors associated with cervical cancer survival. The NCCN suggested that high-grade tumors, lymphovascular invasion (LVI), deep stromal invasion (DSI), parametrial extension, bulky tumors, and positive surgical margins increase the risk of tumor recurrence, distant metastases, and death.^11-13^

 The International Federation of Gynecology and Obstetrics (FIGO) updated the cervical cancer staging system in 2018; in addition, extensive progress has been made in both external and internal radiotherapy (brachytherapy) methods in recent years. This context highlights a perpetual need for up-to-date studies on the treatment outcomes and survival of patients with cervical cancer. This investigation was performed in an academic center where patients with different diagnostic stages from all over the country are referred to for receiving radiotherapy as primary or part of their treatment. The present study aimed to assess the overall survival (OS) and event-free survival (EFS), along with its related factors, such as stage, histopathological features of the tumor, and the type of treatment. Having an estimate of the survival rate of these patients can be an essential step in identifying the existing issues for health system policymakers.

## Materials and Methods

###  Data management

 In this retrospective cohort study, we extracted data by reviewing the records of patients with cervical cancer. The inclusion criteria were all patients diagnosed with cervical cancer who received radiotherapy as a part of their treatment from 1 January 2014 to 31 December 2020 in the Radiation Oncology Department of the Cancer Institute of Imam Khomeini Hospital Complex, Tehran, Iran. Data including age, time of cancer diagnosis, tumor histopathology, type of treatment, tumor stage, tumor size, lymph node involvement, neurovascular, parametrial or stromal invasion, and the patients’ status in the last follow-up were retrieved.

 The information was obtained via a phone call for patients with missing follow-up visits or any incomprehensible medical records. The patients were excluded from the analysis if outcome status could not be acquired. The diagnosis methods of cervical cancer were colposcopy and biopsy (core needle biopsy and cold knife cone biopsy, also called conization), depending on the gynecologist surgeon’s opinion. In the case of patients with suspicious pathology results, the pathology review board of the Imam Khomeini Cancer Institute was re-established, and all diagnosed cervical cancers were approved before starting the treatment. The tumor staging was based on the FIGO staging system (2018).^14^ According to the NCCN, invasive cancers in the early stages of the disease (IA1, IA2, and IB1) and some small IIA tumors are treated by radical hysterectomy or radiotherapy and locally advanced cancers (IB2 to IVA) are treated with radiotherapy and concurrent chemotherapy.^7-10^ In all patients who underwent surgery, the reported tumor stage was based on pathological rather than clinical staging.

 All patients were treated with a 3-D conformal radiotherapy technique (VARIAN) using 18 megavoltage photon energy. Patients received weekly infusions of Cisplatin 40 mg/m^2^ for concurrent chemotherapy. The external radiation dose was 49.1 ± 2.5 (mean ± standard deviation) Grays (Gy). After the completion of radiotherapy, patients underwent high-dose-rate intravaginal brachytherapy to reach the dose requirements of the high-risk clinical target volume if indicated. The mean brachytherapy dose was 16.8 ± 11.9 Gy. The duration of follow-up was 36 months (median). OS was defined as the duration between the first day of radiation therapy and the date of death for any reason or completion of the study. EFS was defined as the initial day of radiation therapy until the date of any clinical or radiologic evidence of loco regional recurrence, metastasis, death, or completion of the study.

###  Statistical analysis

 Descriptive data analysis was used to establish frequencies or mean and standard deviations for categorical and quantitative data, respectively. All confidence intervals (CIs) for parameters to be assessed were made with a significance level of alpha = 0.05 (a 95% confidence level). Kaplan Meyer survival analysis was used to calculate the OS and EFS rates. The log-rank test was used to compare survival between patients receiving radiotherapy as definitive treatment or adjuvant to surgery. In a post hoc analysis, survival data were analyzed based on tumor staging. Cox regression analysis (by the enter method) was used to identify the predictive factors associated with survival. Data were analyzed in SPSS software (version 20). The significance threshold was considered less than 0.05.

###  Ethics statement

 The present study was performed under the tenets of the Declaration of Helsinki. Ethical feasibility was obtained from the Ethics Committee of Tehran University of Medical Sciences (IR.TUMS.IKHC.REC.1399.027).

## Results

 A total of 203 patients were identified, out of whom 187 cases were eventually found eligible to enter the analysis. The mean age of patients at diagnosis was 51.3 ± 13.1 years (range: 22-85), and 63 (34.6%) patients were younger than 45. The histopathology results among patients were as follows: 134 (71.1%) and 33 (18.2%) patients had squamous cell carcinoma (SCC) and adenocarcinoma, respectively, while the other types of histopathology had a lower prevalence (Table 1). At the end of the follow-up period, 32 (17.1%) patients had metastases, among whom 15 (46.9%) cases had multiple bone and visceral metastases. The other sites of metastases had a lower prevalence (Table 1).

**Table 1:** Tumorhistopathology and metastases characteristics in all patients

**Variables**	**Frequency**	**Percent**
Histopathology		
Squamous cell carcinoma	134	71.1
Adenocarcinoma	33	18.2
Adenosquamous carcinoma	5	2.7
Neuroendocrine tumor	2	1.1
Rhabdomyosarcoma	1	0.5
Gastric type adenocarcinoma	1	0.5
Not specified	11	5.9
Metastases		
Yes	32	17.1
No	155	82.9
Metastases sites		
Multiple bone and visceral	15	46.9
Liver	6	18.7
Lung	5	15.6
Bone	3	9.3
Brain	1	3.1
Colon	1	3.1
Intra-peritoneal seeding	1	3.1

 Among all patients, 79 (42.2%) cases underwent surgical treatment followed by adjuvant radiotherapy, 102 (54.5%) subjects underwent definitive radiotherapy, and 6 (3.3%) patients who had metastatic cancer at the time of diagnosis underwent palliative radiotherapy to control symptoms, such as vaginal bleeding and pain. A number of 127 (67.9%) patients were diagnosed with stage IIB or higher. The results of tumor staging based on the FIGO staging system (2018) in all patients and each treatment group are displayed in Table 2.

**Table 2:** Tumor stage and first post-treatment event in all patients and each treatment group

**Variables**	**All Patients**		**Definitive chemoradiotherapy**		**Surgery and adjuvant radiotherapy**	
	**No.**	**%**	**No.**	**%**	**No.**	**%**
Staging						
IA	1	0.5	0	0	1	1.3
IB	31	16.6	2	2	29	36.7
IIA	23	12.3	6	5.9	17	21.5
IIB	33	17.6	24	23.5	9	11.4
IIIA	5	2.7	5	4.9	0	0
IIIB	5	2.7	4	3.9	1	1.3
IIIC	58	31	42	41.2	16	20.2
IVA	20	10.7	19	18.6	1	1.3
IVB	6	3.2	N/A	N/A	N/A	N/A
Missing	5	2.7	0	0	5	6.3
All	187	100	102	100	79	100
First post treatment event^a^						
Local recurrence	15	8.2	7	6.9	8	10.1
Metastases	21	11.6	17	16.7	4	5.1
Both	5	2.7	3	2.9	2	2.5

^a^First post treatment event: Frequencies are measured among patients without metastatic cancer at the time of diagnosis (181, 102, and 79 patients in the first, second, and third column, respectively).

 In patients who underwent surgery followed by adjuvant radiotherapy, the mean tumor size was 32 ± 16 mm, out of whom 18 (22.8%) patients had tumors larger than 4 cm, 16 (20.3%) cases had lymph node involvement (with the median of 13 dissected lymph nodes, interquartile range (IQR) [9-20]), 49 (62%) cases had LVI, 11 (13.9%) subjects had perineural invasion (PNI), 45 (57%) participants had DSI, and 2 (2.5%) patients had positive surgical margin. In patients who underwent definitive chemoradiotherapy (CRT), the tumor size was 42 ± 22 mm, out of whom 47 (46.1%) patients had tumors larger than 4 cm.

 The patients came for post-treatment visits for a median period of 36 months (IQR: 18-51). The first event after the treatment, either definitive or adjuvant radiotherapy, was retrieved in all patients except the six patients with metastatic cancer who underwent palliative radiotherapy at the time of diagnosis. The frequency of events is illustrated in Table 2. The median of overall and event-free survival were 24 and 18 months, respectively. Sequentially, the 1- and 3- year OS rates were 90% and 72%. The 1- and 3-year EFS rates were 76% and 61%, respectively.

 The overall and event-free survival were measured based on histopathology types and compared using the log-rank test. The SCC tumors had increased OS (3-year OS: 78.2%) compared to non-SCC tumors (3-year OS: 65.4%); however, this result was not statistically significant (hazard ratio [HR]: 0.62, 95% CI: 0.33, 1.16, *P* = 0.13). The same findings were observed for event-free survival. Three-year EFS rates of SCC and non-SCC were 62.7% and 59.8%, respectively (HR: 0.82, 95% CI: 0.47, 1.42, *P* = 0.480).

 Univariate cox regression analysis of non-metastatic patients demonstrated that having stage III or higher and bulky tumor (size > 4 cm) predicted a worse OS while undergoing surgery increased OS. When considering covariates in multivariate regression analysis, including age, tumor histology, stage, grade, and tumor size, only stage III or higher was independently associated with a worse OS. In the subgroup of people who underwent surgery, the Cox regression analysis revealed that having a bulky tumor was an independent predictor of a worse OS, which remained significant in multivariate analysis after adjustment for age, stage, LVI, PNI, and DSI (Table 3).

**Table 3:** Hazard ratio (HR) estimation for prognostic risk factors on survival using the Cox model

**Variable**	**Univariate analysis**		**Multivariate analysis**	
	**HR (95% CI)**	***P*** ** value**	**HR (95% CI)**	***P*** ** value**
All non-metastatic patients (OS)				
Stage ≥ III	3.1 (1.5, 6.5)	0.001	2.7 (1.1, 6.4)	0.025
Bulky tumor	2.5 (1.2, 4.9)	0.006	2.0 (0.9, 4.2)	0.070
Surgery	0.3 (0.19, 0.75)	0.005		
All non-metastatic patients (EFS)				
Stage ≥ III	2.4 (1.4, 4.1)	0.002	2.1 (1.1, 4.1)	0.028
Surgery	0.4 (0.2, 0.8)	0.008	No data	
Bulky tumor	4.6 (1.3, 16.4)	0.020	6.8 (1.3,36.0)	0.030

Multivariate analysis, including age, histology, grade, tumor size, and stage.

**Figure 1:**
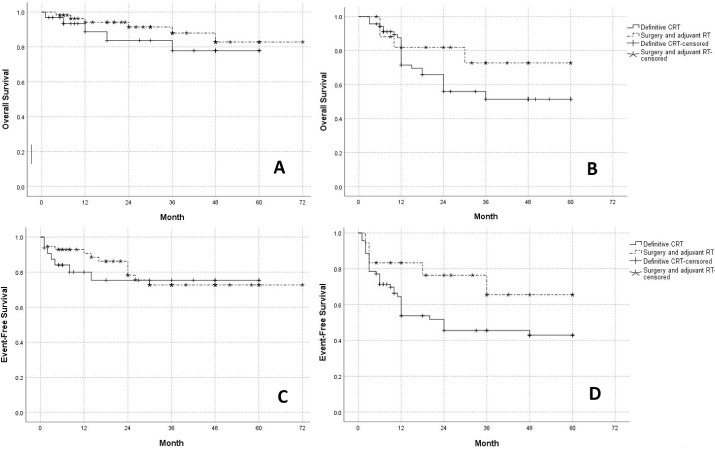
Comparison of survival between different types of treatment with stratification based on the tumor stage. (A) Overall Survival in stages I or II (Log rank: 0.7, *P* = 0.4) (B) Overall Survival in stages III or IVA (Log rank: 1.6, *P =*0.2) (C) Event-Free Survival in stages I or II (Log rank: 0.1*, P =*0.7) (D) Event-Free Survival in stages III or IVA (Log rank: 2.8, *P =*0.1).

 As mentioned earlier, locally-advanced patients are candidates for definitive CRT, while early-staged patients may undergo surgery and adjuvant radiotherapy if indicated. Therefore, the OS and EFS were measured in each treatment group based on the stratification of tumor stage. Log-rank analyses demonstrated that patients with stage I or II treated by surgery vs. those in the same stages receiving CRT had similar OS (Log-rank = 0.7, *P* = 0.4) and EFS (Log-rank = 0.1, *P* = 0.7). Furthermore, among patients with stage III or IVA, the OS (Log-rank = 1.6, *P* = 0.2) and EFS (Log-rank = 2.8, *P* = 0.1) rates were not significantly different between the two treatment groups (Figure 1).

## Discussion

 In the present study, the 1-year and 3-year OS rates were 90% and 72%, respectively. While 1-year and 3- year EFS rates were 76% and 61%, respectively. Several studies have been carried out worldwide to estimate the survival rates and associated prognostic factors in patients with cervical cancer. In China, the 3-year OS of 833 patients with clinical stages IB1 to IV receiving definitive CRT was reported as 84%.^15^ In a multicenter study in Malaysia, the 1-year and 3-year OS rates were 94.1% and 79.3%, respectively; nonetheless, the stated study did not assess factors affecting survival, such as stage, type of treatment, and histopathology.^16^ Due to the lack of an integrated cervical cancer registration system, there are not adequate multi-centric studies with a large sample size on the survival rates of patients with cervical cancer in our country.

 In the studies by Zarchi et al^17^ and Yousefi et al,^18^ the 3-year OS rates in Iranian patients with cervical cancer were obtained at 75.9% and 76.2%, respectively. On the other hand, developed countries, such as Japan, observed a 3-year OS of 94%.^19^ In the present study, the 1-year and 3-year EFS rates were 76% and 61%, respectively. In a study by Fernandez et al. on patients in stages IA1 to IIA1 who were treated surgically, the 3-year disease-free survival was 87%.^20^ In Japan, a recent study reported a high 3-year progression-free survival of 81% among patients with stage IB1-IVA cervical cancer.^19^ The lower overall and event-free survival rates in developing countries can be attributed to the lack of regular screening, the most crucial factor in the early-stage diagnosis of patients with cervical cancer.^1^ A systematic review pointed out that only around half of the Iranian women were aware of cervical cancer, and less than 45% of them had completed at least one-lifetime Pap-smear test.^21^

 Treatment type affects survival rates in some malignancies, such as breast cancer.^22^ The type of treatment in cervical cancer patients is determined based on multiple factors, including the lesion size, disease stage, and comorbidities. In the current study, the OS and EFS were measured in each treatment group based on a stage-matched comparison, and the results did not disclose any statistically significant difference in survival rates. Moreover, although univariate cox regression analysis demonstrated that undergoing surgery increased OS, this result did not remain significant in multivariate analysis after adjusting such factors as tumor size and stage. These findings highlight the importance of stage - not the type of treatment alone - in the survival of patients with cervical cancer. In a similar vein, Landoni et al,^8,23^ in two different randomized trials with 20 years interval and extensive follow-up time, have found that survival rates in stage-matched cervical cancer patients are nearly identical in the surgery and radiotherapy group.

 The results also pointed out that stage III or higher disease and bulky tumor were independently associated with a worse OS. Along the same lines, several studies have highlighted the prognostic role of the tumor stage^24,25^ and tumor size.^26,27^ In agreement with prior studies, in the present research, patients with SCC tumors had better outcomes and survival than other histopathologic types.^28^ In this study, two-thirds of patients were diagnosed with stage IIB or higher. Since this study was conducted in a radiation oncology center where patients are referred for CRT, being in high stages was expected; therefore, the results cannot be generalized to the general population of Iranian patients with cervical cancer.

 In general, the patients who are diagnosed in locally-advanced stages lose the golden time for surgery and undergo radiotherapy, which causes them to experience significant sexual dysfunction, including lack of lubrication, low sexual interest, dyspareunia, and latent adverse effects, such as vaginal stenosis.^29^ A recent investigation on Iranian cancer survivors demonstrated that the patients with cervical cancer had a worse sexual quality of life in terms of psychosexual feelings and worthlessness compared to other gynecological cancers.^30^ Insufficient recorded data about patients’ sexual dysfunction in the present study are indicative of inadequate attention to this critical issue.

 Consistent with previous studies,^1,2^ in the current research, the mean age of patients was 51.3. Patients’ age ranged from 22-85 years, and one-third of them were of reproductive age (younger than 45 years old). According to NCCN, pelvic radiotherapy or chemotherapy may impair the ability of the uterus to bear pregnancy and cause ovarian failure.^31,32^ Despite the importance of infertility in young patients with cervical cancer, there was no documented data on offered fertility preservation methods in our patients’ files.

 In the present study population, the patients came for a post-treatment visit for a median period of 36 months (IQR: 18-51). In a similar vein, in a study conducted by Amouzegar Hashemi et al, 20 years ago, a significant percentage of patients (23.7%) did not come for post-treatment visits, and 43.7% of cases returned for follow-up only for a short time.^33^ It is noteworthy that although the frequency of post-treatment visits has improved compared to the mentioned study, it still does not seem to be sufficient. Accurate post-treatment follow-up indeed results in early diagnosis of recurrence and metastasis; therefore, it is recommended to place emphasis on the importance of regular post-treatment follow-up.

 Left censoring is one of the main limitations of this study. It is possible that the date of recurrence or metastasis, based on the diagnostic test, does not precisely match the date of its occurrence. It is also possible that the date of death in some patients was not recorded exactly in days and was calculated within a one-month interval. The other limitation was the use of one hospital database. Therefore, the results cannot be generalized to Iranian patients with cervical cancer. In the future, multi-centric investigations on a larger population may describe the survival pattern of all cervical cancer patients in Iran. Furthermore, some early-staged patients underwent definitive radiotherapy instead of surgery, perhaps due to the comorbidities that did not allow them to undergo surgery. Moreover, a number of locally-advanced patients underwent surgery in the present study. There are two probable explanations in this regard. Firstly, it might be due to the high rate of patients with non-oncologic surgery referred to our center; moreover, some patients had initially surgical indications based on clinical staging, while after the surgery, the pathology report suggested a higher stage. Further studies are required to evaluate the compatibility of clinical and pathological staging.

## Conclusion

 The extensive progression of cervical cancer treatment methods, especially radiotherapy, has emphasized the need for up-to-date investigations on the survival of patients. Despite the limitations, this study evaluated the survival rates and associated factors of patients with cervical cancer. In the current study, three-year OS and event-free survival rates were lower than those reported in developed countries. Moreover, stage III or higher and bulky tumors were associated with lower survival rates. On the other hand, the type of treatment and histopathology did not significantly impact survival in comparable stages.

**Highlights**

- The median overall and event-free survival rates were 24 and 18 months, respectively.
- The 3- year overall and event-free survival rates were 72% and 61%, respectively.
- The patients came for a post-treatment visit for 36 months.
- Stage III or higher and bulky tumors predicted significantly lower survival.
- Survival was not significantly correlated with treatment or histopathology.

## Conflicts of interest

 The authors have no conflict of interest to declare for this study.

## Funding

 None.
